# The Relation of the Eye to General Disease

**Published:** 1913-01

**Authors:** Cecil Stockard

**Affiliations:** Atlanta, Ga.; 903-5 Empire Life Building


### Journal-Record of Medicine

Successor to Atlanta Medical and Surgical Journal, Established 1855
and Southern Medical Record, Established 1870.

OWNED BY THE ATLANTA MEDICAL JOURNAL CO.

**Published Monthly**

*Official Organ Fulton County Medical Society, State Examining Board, Presbyterian Hospital, Atlanta, Birmingham and Atlantic Railroad Surgeons' Association, Chattahoochee Valley Medical and Surgical Association, Etc.*

EDGAR G. BALLENGER, M. D., Editor.

BERNARD WOLFF, M. D., Supervising Editor.

A. W. STIRLING, M. D., C. M., D. P. H., J. S. HURT, B. Ph., M. D.

GEO. M. NILES, M. D., W. J. LOVE, M. D., (Ala.); Associate Editors.

E. W. ALLEN, Business Manager.

COLLABORATORS

Dr. W. F. WESTMORLAND, General Surgery.
F. W. McRAE, M. D., Abdominal Surgery.
H. F. HARRIS, M. D., Pathology and Bacteriology.
E. B. BLOCK, M. D., Diseases of the Nervous System.
MICHAEL HOKE, M. D., Orthopedic Surgery.
CYRUS W. STRICKLER, M. D., Legal Medicine and Medical Legislation.
E. C. DAVIS, A. B., M. D., Obstetrics.
E. G. JONES, A. B., M. D., Gynecology.
R. T. DORSEY, Jr., B. S. M. D., Medicine.
L. M. GAINES, A. B., M. D., Internal Medicine.
GEO. C. MIZELL, M. D., Diseases of the Stomach and Intestines.
L. B. CLARKE, M. D., Pediatrics.
EDGAR PAULIN, M. D., Opsonic Medicine.
THEODORE TOEPEL, M. D., Mechano Therapy.
R. R. DALY, M. D., Medical Society.
A. W. STIRLING, M. D., etc., Diseases of the Eye, Ear, Nose and Throat.
BERNARD WOLFF, M. D., Diseases of the Skin.
E. G. BALLENGER, M. D., Diseases of the Genito-Urinary Organs.

VOL. LIX. JANUARY 1913 No. 10

### THE RELATION OF THE EYE TO GENERAL DISEASE

By Cecil Stockard, M. D., Atlanta, Ga.

Several states have, in recent years, passed bills to regulate and license the practice of so-called optometry, with the avowed intention of preventing incompetent men from traveling over the country fitting glasses upon as many people as will buy them, and then going on to the next town to repeat the performance; and so far it is a good thing, but the public do not distinguish between "optometrist" and "oculist," and are apt to think that opticians are licensed by the state as competent to treat all manner of eye conditions.

It is my purpose in this paper to sketch briefly some of the conditions which are apt to confront an eye-man in which a general knowledge of medicine is essential.

Perhaps the most common are those in which indicanuria is associated with a refractive error. In these cases it is necessary to correct both conditions, that of the eyes, and the digestive disturbances before the headache, eye strain, etc., will disappear.

Indicanuria seems frequently to cause a disturbance of the extraocular muscles, occasionally even a spastic squint, and also occasionally an impairment of vision due probably to retrobulbar neuritis.

So important is role played by syphilis in eye troubles that it should be considered as a possible cause in practically every inflammatory condition, especially iritis, keratitis, chorioditis, aptic atrophy, neuroretinitis and various paralyses.

Rheumatism is also a frequent cause of iritis, as well as some other inflammatory conditions, such as episcleritis, etc.

Gonorrhoeal ophthalmia is, of course, a secondary infection, and scarcely comes under the scope of this paper.

Brights disease is one of the most important to the ophthalmologist because the retina often shows the first signs of the disease. This is also true in the alubuminuria of pregnancy, and in these cases recovery usually follows abortion.

Cataracts are frequently found associated with diabetes, as are occasionally retinitis and other serious conditions. Various anaemias give rise to ocular change, such as neuro-retinitis, asthenopia, and the cataracts and other changes of Hookworm diseases as described by Dr. Calhoun.

Consanguinity of parents frequently gives rise to retinitis pigmentosa, though I believe that for some reason this condition is extremely rare in the negro.

Gynecological conditions are frequently associated with ocular symptoms, chiefly myasthenia, and functional disturbances of the retina, though occasionally more serious complications occur. Enlarged prostates and urethral affections are at times associated with similar conditions.

In hysteria, in the eye as elsewhere, almost any subjective symptoms may occur, pains, temporary blindness, and also muscular disturbances, dilated pupils, etc.

The teeth, gastro-intestinal tract, respiratory tract, vascular system, exophthalmic goitre, lymphatic glands, aural disease, adjacent sinuses and diseases of the skin must also be borne in mind in connection with the various ocular affections.

Tuberculosis occasionlly affects the eyes,, and scrofulous children are practically prone to inflammatory conditions of the cornea, iris, etc.

Meningitis may produce ocular changes of a most distressing type.

Neuro-retinitis may result from a large dose of quinine, a number of cases of neuritis have been reported following the administration of salvarsan. Alcohol and tobacco amblyopias are fairly common.

Decreased sensitiveness to pressure on the eyeball is said to be sometimes a symptom of beginning tabes.

Irregularity in the size of the pupils has been observed in incipient tuberculosis.

There is another important side to the question and that is, general or extra-ocular symptoms caused by ocular disturbances,

Under this head may be included the referred pains of iritis and glaucoma, but the principal conditions are those caused by refractive errors.

Myopia, or near-sightedness, rarely causes headaches, eye-strain or conjunctivitis. These patients however, are apt to be studious, and rarely enjoy out-of-door sports, because they do not see well enough to at distance, and also the light hurts their eyes as it is not properly focused on the retina.

Hyperopia or farsightedness and also astigmatism, on the contrary, commonly cause conjunctivitis, eye strain, and headaches. These headaches vary very much in location and degree, usually come on after close work and are sometimes accompanied by nausea, especially where there is astigmatism.

Errors of refraction are the usual causes of strabismus or crossed eyes, the eye usually diverges or turns outward in myopia, and converges or turns inward in hyperopia. It should be remembered that measles, whooping cough, scarlet fever, etc., do not cause strabismus, as parents frequently insist.

I have taken a very general view of this subject with the idea of emphasizing its importance, and showing that ophthalmology should never be considered as a separate profession, but as a more or less important branch of medicine.

903-5 Empire Life Building.

